# The impact of COVID-19 on TB: a review of the data

**DOI:** 10.5588/ijtld.21.0148

**Published:** 2021-06-01

**Authors:** C. F. McQuaid, A. Vassall, T. Cohen, K. Fiekert, R. G. White

**Affiliations:** 1 TB Modelling Group, TB Centre and Centre for Mathematical Modelling of Infectious Diseases, Department of Infectious Disease Epidemiology, Faculty of Epidemiology and Population Health, London School of Hygiene & Tropical Medicine (LSHTM), London, UK; 2 Department of Global Health Development, Faculty of Public Health and Policy, LSHTM, London, UK; 3 Yale School of Public Health, Laboratory of Epidemiology and Public Health, New Haven, CT, USA; 4 KNCV Tuberculosefonds, The Hague, the Netherlands

**Keywords:** TB, health services, vulnerability, transmission, resource

## Abstract

Early in the COVID-19 pandemic, models predicted hundreds of thousands of additional TB deaths as a result of health service disruption. To date, empirical evidence on the effects of COVID-19 on TB outcomes has been limited. Here we summarise the evidence available at a country level, identifying broad mechanisms by which COVID-19 may modify TB burden and mitigation efforts. From the data, it is clear that there have been substantial disruptions to TB health services and an increase in vulnerability to TB. Evidence for changes in *Mycobacterium tuberculosis* transmission is limited, and it remains unclear how the resources required and available for the TB response have changed. To advocate for additional funding to mitigate the impact of COVID-19 on the global TB burden, and to efficiently allocate resources for the TB response, requires a significant improvement in the TB data available.

## Abstract (FR)

Au début de la pandémie de COVID-19, les modèles prévoyaient des centaines de milliers de décès supplémentaires dus à la TB en raison des perturbations des services de santé. Jusqu’à présent, les preuves empiriques des effets de COVID-19 sur les résultats de la TB ont été limitées. Nous résumons ici les preuves disponibles au niveau national, en identifiant les mécanismes généraux par lesquels COVID-19 pourrait modifier la charge de la TB et les efforts d’atténuation. Lorsque les données sont disponibles, il est clair qu’il y a eu d’importantes perturbations des services de santé de la TB et une augmentation de la vulnérabilité à la TB. Les preuves de changements dans la transmission de *Mycobacterium tuberculosis* sont limitées, et l’évolution des ressources nécessaires et disponibles pour la réponse à la TB n’est pas claire. Une amélioration significative des données sur la TB est nécessaire pour obtenir des fonds supplémentaires afin d’atténuer l’impact de COVID-19 sur le fardeau mondial de la TB et pour allouer efficacement les ressources à la réponse à la TB.

GIVEN CONCERNS FOR MAINTAINING TB CARE and prevention services during the COVID-19 pandemic,^1^ mathematical modellers have attempted to estimate the potential impact on TB incidence and mortality.^2^–^5^ Despite the use of different methods and assumptions about the future of the pandemic, as well as modelling for a variety of settings (including India, China, South Africa, Kenya, Ukraine and Brazil), these analyses reached broadly similar conclusions. Specifically, TB incidence, and especially TB mortality, are projected to increase by around 5–15% over the next 5 years, amounting to hundreds of thousands of additional TB deaths worldwide. Indeed, the WHO now estimates that half a million more people may have died from TB in 2020 alone.^6^ These early modelling analyses, however, relied on a number of assumptions, which should ideally be reevaluated in the context of empirical data. Since these analyses were produced, little evidence has been systematically collected to quantify the impact of COVID-19 on TB burden. A data-driven understanding of this impact is necessary to support efforts to mitigate it, revise the implementation of TB services and allocate resources to different TB interventions. To implement and prioritise effectively, it is essential to understand the current situation.

We expect COVID-19 to affect TB outcomes differently by setting. For example, countries with a large TB burden, such as India and Viet Nam, have experienced very different COVID-19 incidence.^7^ Countries with a similar COVID-19 burden, such as Brazil and Argentina, have experienced different levels of health system disruption.^8^ Indeed, within individual countries the impact will further vary between rural and urban areas, by socio-economic status, and as response measures vary spatially. With all of this variation, it is therefore vital to focus on the measurement of setting-specific impact. It is also important to identify when the impact was measured, as the temporal effect of the pandemic varies between countries.

Here we review the evidence available, to inform how the implementation and allocation of resources by TB programmes could be revised. We identify where country-specific data and evidence can be found to quantify the impact of COVID-19 on TB outcomes, and the costs of any mitigation. In Figure 1, we outline the conceptual framework for our narrative review, specifying how COVID-19 may impact across the TB care cascade, identifying disruption to TB health service delivery and changes in demand, alterations in vulnerability to TB (including comorbidities and risk factors) and opportunities for *Mycobacterium tuberculosis* transmission. We then identify data on the impact of COVID-19 on both availability and requirements of TB resources, and collate this evidence in the Table. We end by highlighting knowledge gaps that should be prioritised for study.

**Table:** Available or upcoming data on the impact of COVID-19 on TB by country for WHO high TB, TB-HIV and multidrug-resistant TB burden countries^13^

Country	Health services data							Vulnerability data			Transmission data					Resource data		
			
	Diagnosis			Treatment		Prevention		HIV		Poverty	No control measures		Under control measures			Required		Available
								
	Cases	Testing	DST	Delays	Outcomes	BCG coverage	Preventive therapy	Testing	ART	Patient costs	Household transmission	Contacts	Contacts	Mobility	Mask-wearing	Resource utilisation	Prices	Budgets
Angola	32											76		97	99			118
Azerbaijan												76			99			118
Bangladesh	32									13	107	76		97	99			118
Belarus	32											76		97	99			118
Botswana								70	70		107	76		97	99			118
Brazil	23,25,32	44		44	44		25	70		13	107,108	76		97	99			
Cambodia	13,32							70				76		97	99			
Cameroon									70	13		76		97	99			118
Central African Republic												76			99			118
Chad												76			99			118
China	13–17,30	15–17		17	16,17							76,77,80,81	94		100			
Congo												76			99			118
DPR Korea	32											76			99			
DR Congo	32											76			99			118
Eswatini												76			99			118
Ethiopia	32				46			70	70	13		76			99			118
Ghana												76		97	99			118
Guinea-Bissau											107	76		97	99			
India	13,20,21–25,27, 28 31,32,35					31,51				13	107	76,77		97	100			118
Indonesia	13,32							70	70	13	107	76		97	100			
Kazakhstan	32											76		97	99			118
Kenya	13,24,25,32	43	43		24		25	24,70	70		104,107	76,82,83	95	97	99			118
Kyrgyzstan	32								70			76		97	99			118
Lesotho	32							70	70			76			99			118
Liberia								70				76			99			118
Malawi	24,34	43	43		24			24		13	104,109	76,84			99			118
Mozambique	13,32							70	70	13		76		97	99			118
Myanmar								70	70			76		97	99			118
Namibia	13,32									13		76		97	99			118
Nigeria	18,32	40							70		107	76		97	99			118
Pakistan	22,32,33				45	52 53						76		97	99			118
Papua New Guinea	32											76		97	99			118
Peru	32							70	70	13	107,110,111	76,85		97	99			118
Philippines	13,25,29 32	41	41				41					76		97	100			118
Republic of Moldova	32											76		97	99			118
Russian Federation	25,32						25					76,77,86,87			99			
Sierra Leone	13,19,25 32						25	70	70		107	76			99			
Somalia															99			118
South Africa	13,26,32	42	42	47			49	70	70	13	104,107,112–115	76,77,88–90	96,119	97	99			118
Tajikistan	32					70	70			76	97	99						
Tanzania	13,32					70				76	97	99						
Thailand	13,32							13		76,78	97	100						
Uganda	32,120	44		44		70			104,107,108,116	76,91	97	99	118					
Ukraine	32					70	70			76	97	99	118					
Uzbekistan										76		99	118					
Viet Nam	13,32	44		44					107,117	76,92	97	100	118					
Zambia	13,32				50			13		76,89,90	97	99	118					
Zimbabwe	24	43	43	24		24,70	70			76,93	97	99	118					

DST = drug susceptibility testing; BCG = bacilli Calmette-Guérin; ART =antiretroviral therapy; DPR = Democratic People’s Republic; DR = Democratic Republic.

**Figure 1.**
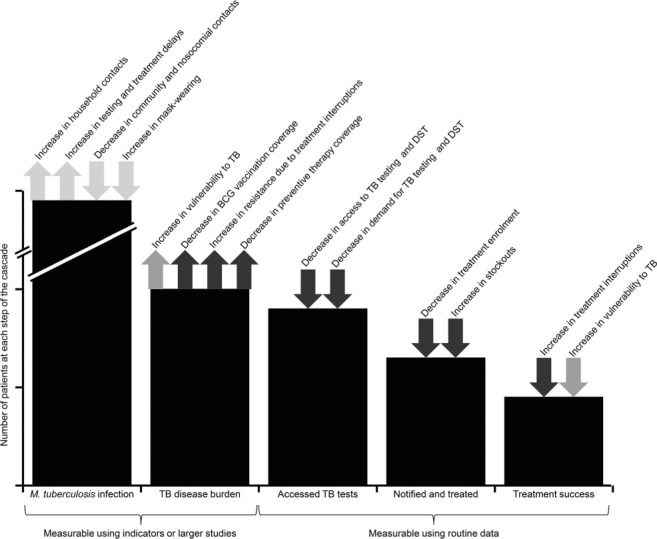
Potential impact of COVID-19 on the TB care cascade. Arrows indicate an increase or a decrease in number of patients at that point of the cascade, including the logic behind the change. black arrows indicate an impact on health service delivery and demand, grey arrows indicate an impact on vulnerability to TB, and light grey arrows indicate an impact on M. tuberculosis transmission. BCG = bacilli Calmette-Guérin; DST = drug susceptibility testing.

## SEARCH STRATEGY AND SELECTION CRITERIA

We conducted a narrative and bibliometric review, combining a rapid semi-systematic search and convening a range of experts. For the rapid review, references were identified through searches of PubMed, medRxiv and bioRxiv for articles published from January 2020 to March 2021, using the terms “COVID” or “SARS” or “corona”, and “TB” or “tuberculosis”. In addition, literature relevant to TB vulnerabilities, *Mycobacterium tuberculosis* transmission and TB resources was identified through the authors’ personal libraries. Additional relevant grey literature was identified through communication with the WHO Global TB Department, as well as through a virtual meeting of the TB Modelling and Analysis Consortium, where a group of TB experts from global agencies, academic institutions and country programmes were invited to identify additional sources of data and to confirm and highlight priority knowledge gaps. Grey literature was included in this instance as it represents a significant proportion of the relevant data available to country-level TB decision makers when making policy choices. Articles resulting from these searches and relevant references cited in those articles were reviewed.

Articles which contained data on country-specific quantitative changes to TB health service indicators, burden of TB vulnerabilities, *M. tuberculosis* transmission and TB resources for the WHO high TB, TBHIV and multidrug-resistant TB (MDR-TB) burden countries were included, and data extracted from these articles. A summary of sources found by country on each topic is presented in the Table. We provide a narrative synthesis of our findings below.

Ethical approval was not required for this study as this was a review of existing studies.

## TB HEALTH SERVICES

The provision of TB health services (TB diagnosis, care and prevention services), and access to these services, has been severely disrupted by COVID-19.^9^–^11^ TB service providers across many high TB burden contexts have faced difficulties in service provision, due to lack of appropriate equipment and capacity, restrictions to movement (affecting health care workers, commodities and stock) and reallocation of resources.^10^ Meanwhile, individual TB patients have struggled to access TB services, either through fear of SARS-CoV-2 infection, fear of stigma, restrictions to movement, reduced health facility opening hours or reductions in the ability to pay for care or transport.^9^ Globally, TB diagnosis, care and prevention has been affected as a result. However, nearly a year after these disruptions began, relatively little high-level information is available, focused primarily on reductions in the number of TB patients.^12^ Most data that are available deal with the first two quarters of 2020, with little data except for patient numbers available for quarters three and four when services might be expected to be somewhat restored.

Most high TB burden countries have observed some changes in TB case numbers or notifications (when TB is diagnosed in a patient and this is reported through the national surveillance system) that have resulted due to COVID-19.^13^–^35^ Continuous surveillance systems and current data collection efforts^36^,^37^ suggest that additional data may also be forthcoming. In general, TB notifications decreased significantly during the early stages of the pandemic compared to previous years. The United States Agency for International Development (USAID) preliminarily estimates are that over 1 million fewer cases in 24 high TB burden countries alone may have been notified in 2020 as a result of the pandemic, with a 7% relative reduction in Africa, a 15% reduction in Central Asia and Europe, and a 27% reduction in Asia compared to 2019.^38^ More recent estimates by the WHO,^6^ the Global Fund to Fight AIDS, Tuberculosis and Malaria (the Global Fund)^39^ and the Stop TB partnership^35^ suggest that globally around 20–30% fewer people were notified with TB than in 2019, with 45% fewer tested for MDR-TB. A limited number of countries appear to have either avoided this trend (such as Mozambique and Tanzania) or have seen notifications dip and since recover to pre-pandemic levels (such as China and Viet Nam).^13^ However, without data on TB testing and positivity rates it is difficult to determine whether this widespread decrease in notifications reflects a true decrease in incidence, or a decrease in access to TB diagnostic services. In several countries where testing data, including for drug susceptibility testing, are available (China,^15^–^17^ Nigeria,^40^ the Philippines^41^ and South Africa,^42^ with further studies underway in Kenya, Malawi and Zimbabwe,^43^ as well as Brazil, Uganda and Viet Nam^44^), testing decreased. In South Africa, this was accompanied by a corresponding increase in the proportion of TB tests that were positive.^42^ The implication of this is that there are likely to be large numbers of undiagnosed cases of TB in the community, who may now face poorer treatment outcomes due to delayed diagnosis and treatment.

In addition to reducing TB diagnosis, COVID-19 may have hampered treatment for TB patients due to limited treatment support and medication stockouts. Such disruption could increase the risk of treatment interruption and delay, and decrease treatment adherence, which can be expected to result in worsening TB treatment outcomes. Due to the long duration of TB treatment, definitive data on changes in TB treatment outcomes as a result of COVID-19 may not be available for several months. In brief reports of patients in private-sector centres in Pakistan,^45^ a Chinese province^16^ and cities in Ethiopia^46^ and Zimbabwe,^24^ treatment outcomes and support have worsened slightly (approximately 5–15% relative reduction). On the other hand, analysis of data from China^17^ and of a small number of patients in cities in Kenya and Malawi^24^ did not show strong evidence of a significant reduction in treatment success. Also, non-TB-specific data in a South African province showed that numbers of clinic visits in general did not decline, although there was a significant (but temporary) decrease in child healthcare visits.^47^ Further studies are underway in Brazil, Uganda and Viet Nam.^44^ At this point, it is difficult to determine how effective calls for the use of digital technologies, additional medicines to take home and other approaches to ensure adequate treatment^48^ have been, although many patients have reported feeling insufficiently supported.^9^

TB prevention services such as routine bacilli Calmette-Guérin (BCG) vaccination, household contact management and preventive therapy are also likely to have been impacted by the COVID-19 pandemic. Routine reporting on these indicators is limited, and this challenges efforts to quantify the impact of COVID-19 on provision of these preventive services. TB centres in Brazil,^25^ Kenya,^25^ the Philippines,^41^ Russia,^25^ South Africa,^49^ Sierra Leone^25^ and Zambia^50^ reported relative declines in preventive therapy enrolment of 30–70%, although in the Philippines this decline appears to be consistent with pre-pandemic recent trends, and in South Africa as well as one Brazilian centre, preventive therapy enrolment seems to have recovered to pre-COVID levels. Meanwhile, India^31^,^51^ and Pakistan^52^,^53^ reported major decreases in relative BCG vaccination coverage of up to 60%, with significant potential consequences for paediatric TB mortality in particular.^54^

## VULNERABILITY TO TB

Just as the COVID-19 pandemic has impacted TB burden, it has also impacted global vulnerability to TB, through a general decrease in health care access, an increase in poverty and the potential for post-COVID-19 lung disease. These vulnerabilities could increase progression to TB disease among those with *M. tuberculosis* infection, as well as worsen treatment outcomes for patients on treatment. Modelling evidence broadly suggests that an increase in these vulnerabilities is likely,^4^,^55^,^56^ but clear evidence of an increase is thus far scarce.

There is growing evidence to suggest that previous or current TB infection or disease are associated with poor COVID-19 outcomes,^57^–^60^ including an approximately two- to three-fold increase in mortality (which occurred more quickly) and a 25% relative decrease in the possibility of recovery (which occurred more slowly) for COVID-19 coinfection with current TB disease.^61^–^64^ However, while there is little evidence as yet that previous SARS-CoV-2 infection or COVID-19 disease affect either progression to TB disease or TB treatment outcomes,^65^ the possibility of post-COVID-19 lung damage and subsequent vulnerability to TB is a major concern.^12^,^58^,^66^ A number of studies are underway to investigate this issue.^67^–^69^

At the same time, a similar decrease in health care provision to that described above for TB could significantly impact TB vulnerabilities such as HIV and diabetes. Data for HIV health services are available from UNAIDS for many,^70^ but not all, high TB-HIV burden countries. This includes both testing and treatment data for Botswana, Ethiopia, Indonesia, Kenya, Lesotho, Mozambique, Myanmar, Peru, Sierra Leone, Tajikistan, Ukraine and Zimbabwe, testing data only for Brazil, Cambodia, Liberia, Uganda and Tanzania, (as well as the capital cities of Kenya, Malawi and Zimbabwe^24^) and treatment data only for Cameroon, Kyrgyzstan and Nigeria. Broadly, HIV testing has declined significantly due to COVID-19, particularly in the early stages of the pandemic. However, in many settings this has recovered somewhat, through HIV self-testing.^70^ In addition, the proportion of tests that are positive has generally not changed, suggesting that there has likely been relative stability in testing practices, if not coverage. Meanwhile, although numbers on treatment have been less affected, numbers initiating treatment have declined precipitously and generally not returned to pre-COVID-19 levels.^70^ However, it is not yet clear how the actual burden of HIV, diabetes and other TB vulnerabilities has increased due to COVID-19.

Poverty is expected to increase due to COVID-19,^55^ and surveys show it is driving people with TB into poverty and increasing inequities.^9^ Although data on changes to costs faced by TB patients are not yet available, national surveys are already underway or planned in 13 of the 48 high TB, TB-HIV or MDRTB burden countries.^13^ In particular, one survey recently completed in India contains samples from both pre- and mid-pandemic periods. The effects of an increase in poverty and inequality include a likely increase in catastrophic costs (>20% of household annual income) faced by TB patients and a resulting inability to access TB health services as discussed above.^71^ Increases in poor living conditions and malnutrition can also drive increases in TB.^72^,^73^ With as much as 30–50% of TB incidence attributable to malnutrition, the potential longer-term consequences for these economic effects on the TB epidemic will be important to investigate.^74^

## *MYCOBACTERIUM TUBERCULOSIS* TRANSMISSION

We do not yet know how *M. tuberculosis* transmission has been affected by COVID-19 and the use of interventions to reduce SARS-CoV-2 transmission. A reduction in respiratory contacts in the community and healthcare settings, in addition to the widespread use of masks, may reduce transmission of *M. tuberculosis*, as has been observed for influenza.^75^ However, a potential increase in contact within household settings, and the long duration of latent TB infection and TB disease as compared to COVID-19, may increase transmission in these settings. This effect could be compounded if decreasing access to TB health services increases the duration of TB infectiousness and increasing vulnerabilities lead to greater risk of TB disease.

Studying TB transmission is challenging. One approach to estimate potential changes in *M. tuberculosis* transmission is to consider changes in contacts in different social settings over time, particularly as these data are collected elsewhere to understand changes to SARS-CoV-2 transmission. Unfortunately, for most high TB burden countries, contact surveys are limited. While synthetic contact matrices are available for all high TB burden countries except Somalia,^76^–^79^ only 10 high TB, TB-HIV or MDR-TB burden countries have contact surveys available from before the pandemic.^80^–^93^ Furthermore, only China,^94^ Kenya^95^ and South Africa^96^ have contact surveys available from during the pandemic (with a survey currently underway in Pakistan), showing a marked decrease in contacts outside of the household.

New sources of mobility data, for example, from Google^97^ or mobile phone providers, suggest massive, time-varying changes in population movements as a result of COVID-19. Although this does not provide information on how contacts have changed, it does allow for a better understanding of locations (such as public transport or places of worship), where contacts have decreased. This can be used, alongside contact surveys where the location of contact was recorded, to estimate likely reductions in contacts. A major caveat is that those surveyed include mobile phone owners only, which may underrepresent both TB patients^98^ and potentially those unable to practice physical distancing.

As a result of efforts to understand the pandemic, data on mask-wearing are widely available for all high TB burden countries, and shows a major increase,^99^,^100^ which has the potential to be of great benefit to the TB response.^101^ Although the impact of mask use on *M. tuberculosis* transmission is poorly understood,^102^ it may be significant in some settings, particularly if sustained for significant time periods.^103^

The impact on *M. tuberculosis* transmission of changes in contacts or mask-wearing in particular locations is dependent on the extent to which transmission occurs in those locations and the potential for changes in per-contact risk to affect overall risk of transmission. Studies from before the pandemic suggest that even for children only 10–30% of population-attributable transmission is due to household exposure.^104^,^105^ Presuming contact saturation within the home limits the amount of additional transmission that could occur as a result of increased time spent there,^106^ decreased community contact and mask-wearing could significantly reduce overall *M. tuberculosis* transmission per person with TB disease. The relative importance of this reduction in community transmission is likely to be dependent on the extent to which transmission occurs outside of the home. Some evidence of the proportion of *M. tuberculosis* transmission attributable to the household or other locations is available for a number of countries, where this may depend in part on the burden of disease.^104^,^107^–^117^

## TB RESOURCES

To understand and mitigate the consequences of COVID-19 on TB interventions and outcomes, it is necessary to understand how the resource needs of TB services have changed, and the impact of COVID-19 on the resources available. First, approaches to delivering TB interventions are likely to have changed, either through design (such as an increased need for personal protective equipment, or additional staff time required for infection control and physical distancing measures), or through shortages or constraints to some inputs (such as staffing and diagnostic capacity).^48^ Second, prices for different intervention inputs could change substantially as demand increases. Third, the costs of providing services are linked to service volumes (for example, a short-term reduction in demand may result in temporary over capacity of some TB focused resources). Finally, the available budget for supporting TB services may be lower, with resources diverted to COVID-19 care or mitigation. Indeed, nearly half of high TB burden countries reported reallocation of TB funding to the COVID-19 response,^13^ with TB funding decreasing significantly.^9^ Although additional funding to many countries (apart from Brazil, Cambodia, China, DPR Korea, Guinea-Bissau, Indonesia, Russian Federation, Sierra Leone, Tajikistan, Thailand and Tanzania) has been made available (e.g., by funders such as the Global Fund),^118^ this is aimed at mitigating the impact on the HIV, TB and malaria programmes in general, and does not shed light on any changes to the budget available to the TB programme. We found no country-level quantitative data currently publicly available on the impact of COVID-19 on the resources available to (or required for) the TB response. During the expert meeting, researchers confirmed that in the main, cost data collection had been suspended during the COVID-19 period.

## CONCLUSION

In general, where data are available, TB health services appear to have decreased significantly in most settings due to COVID-19. Numbers of patients, as well as testing and prevention coverage, have decreased more noticeably than treatment outcomes, although few data are available on the latter. Ensuring adequate treatment for known TB patients, through provision of additional medicine and digital treatment support, appears to be more amenable to physical distancing than TB diagnosis, which typically requires direct contact between individuals. Meanwhile, vulnerability to TB has widely increased. HIV services appear to have recovered somewhat, although the potential for COVID-19-related lung damage to lead to widespread vulnerability to TB is still unknown, as are the impacts of changes in other vulnerabilities such as diabetes and malnutrition. Data on the impact of an increase in poverty on TB patient costs are currently unavailable, although many studies are underway to address this. Unlike TB health services, which have in a number of cases been restored, vulnerabilities are likely to continue to increase despite COVID-19 vaccines being available, as widespread poverty remains and SARS-CoV-2 infections continue to increase. Although community transmission of *M. tuberculosis* has likely decreased, the effect of household transmission and a potential increase in cases means that it is difficult to draw any conclusions on changes in *M. tuberculosis* transmission. Indeed, this may never be possible, although the location of transmission events is likely to have shifted. Finally, while some additional funding has been allocated by global agencies to countries for their TB response, it remains unclear how overall health system resource constraints and the changing resources of service delivery are impacting TB. Although it is difficult to draw any conclusions on the geographic availability of data, we note that little appear to be available for the high MDR-TB burden countries of Central Asia, while many smaller studies are available for countries in sub-Saharan Africa. In general, only a limited number of countries (such as China and South Africa) have good data available across a range of indicators.

**Figure 2.**
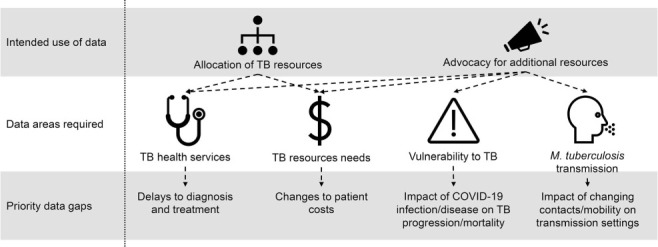
Outline of priority gaps that remain for understanding and mitigating the impact of COVID-19 on TB.

When identifying priority gaps that remain for understanding and mitigating the impact of COVID-19 on TB, it is important to be clear on what these data will be used for. We suggest that this should primarily be to allocate TB resources more efficiently and to help advocate for additional resources for the TB response. The first of these requires a good understanding of the effect on health services, and the resources available and required to restore these to at least pre-pandemic levels. In addition, the second point requires an understanding of how vulnerability to TB and *M. tuberculosis* transmission have changed. In an online meeting of 60 TB experts (TB Modelling and Analysis Consortium meeting on the impact and mitigation of COVID-19 on TB, held on 12 January 2021), a range of priorities were identified from across the four broad areas identified above and these are outlined in Figure 2. There was strong support for data on delays to diagnosis and treatment, changes to patient costs of TB services, the impact of COVID-19 infection and disease on vulnerability to TB and mortality, and the effect of changing contacts and mobility on household and community transmission of *M. tuberculosis*. A key priority was the longer-term requirement for more responsive TB information systems. While this has not been as much of a problem in the past, the rapid nature of the COVID-19 pandemic has highlighted the need for frequently reported, disaggregated TB health service availability and use data, to allow for an appropriate response. A lack of real-time data to make decisions suggests that investment in a change to TB information and reporting systems to enhance real-time empirical evidence (as can be seen for COVID-19) is required. Data collation and monitoring efforts, by an appropriate global stakeholder, should additionally be strengthened.

In conclusion, while the numbers of TB patients have declined globally, it is not yet possible to determine the key causes for these declines, what they represent in terms of changing TB burden and what action is required to mitigate this. Advocating for additional funding to mitigate the impact of COVID-19 on the global TB burden, and allocating available resources efficiently for the TB response, will require a significant improvement in the availability of TB data.
